# Histidine-Rich Glycoprotein Protects from Systemic *Candida* Infection

**DOI:** 10.1371/journal.ppat.1000116

**Published:** 2008-08-01

**Authors:** Victoria Rydengård, Oonagh Shannon, Katarina Lundqvist, Lukasz Kacprzyk, Anna Chalupka, Anna-Karin Olsson, Matthias Mörgelin, Willi Jahnen-Dechent, Martin Malmsten, Artur Schmidtchen

**Affiliations:** 1 Section of Dermatology and Venereology, Department of Clinical Sciences, Lund University, Biomedical Center, Lund, Sweden; 2 Section of Infection Medicine, Department of Clinical Sciences, Lund University, Biomedical Center, Lund, Sweden; 3 Department of Microbiology, Faculty of Biochemistry, Biophysics & Biotechnology, Jagiellonian University, Kraków, Poland; 4 Department of Medical Biochemistry and Microbiology, Uppsala University, Uppsala Biomedical Center, Uppsala, Sweden; 5 Department of Biomedical Engineering, RWTH Aachen University Hospital, Aachen, Germany; 6 Department of Pharmacy, Uppsala University, Uppsala Biomedical Center, Uppsala, Sweden; Carnegie Mellon University, United States of America

## Abstract

Fungi, such as *Candida* spp., are commonly found on the skin and at mucosal surfaces. Yet, they rarely cause invasive infections in immunocompetent individuals, an observation reflecting the ability of our innate immune system to control potentially invasive microbes found at biological boundaries. Antimicrobial proteins and peptides are becoming increasingly recognized as important effectors of innate immunity. This is illustrated further by the present investigation, demonstrating a novel antifungal role of histidine-rich glycoprotein (HRG), an abundant and multimodular plasma protein. HRG bound to *Candida* cells, and induced breaks in the cell walls of the organisms. Correspondingly, HRG preferentially lysed ergosterol-containing liposomes but not cholesterol-containing ones, indicating a specificity for fungal versus other types of eukaryotic membranes. Both antifungal and membrane-rupturing activities of HRG were enhanced at low pH, and mapped to the histidine-rich region of the protein. *Ex vivo*, HRG-containing plasma as well as fibrin clots exerted antifungal effects. *In vivo*, Hrg^−/−^ mice were susceptible to infection by *C. albicans*, in contrast to wild-type mice, which were highly resistant to infection. The results demonstrate a key and previously unknown antifungal role of HRG in innate immunity.

## Author Summary

It has been estimated that humans contain about 1 kg of microbes, an observation that reflects our coexistence with colonizing microbes such as bacteria and fungi. The fungal species *Candida* is present as a commensal at mucosal surfaces and on skin. Although it may cause life-threatening infections, such as sepsis, particularly in immunocompromised individuals, it seldom causes disease in normal individuals. In order to control our microbial flora, humans as well as virtually all life forms are armoured with various proteins and peptides that comprise integral parts of our innate immune system. Here we describe a new component in this system; histidine-rich glycoprotein (HRG), an abundant plasma protein. We show, using a combination of microbiological, biochemical, and biophysical methods, that HRG exerts a potent antifungal activity, which is mediated via a histidine-rich region of the protein, and targets ergosterol-rich membrane structures such as those of *Candida*. HRG killed *Candida* both in plasma as well as when incorporated into fibrin clots. In mouse infection models, HRG was protective against systemic infection by *Candida*, indicating a novel antifungal role of HRG in innate immunity.

## Introduction

The innate immune system, based on antimicrobial peptides (AMP) and proteins, provides a first line of defence against invading microbes [1]–[3]. At present, over 880 different AMPs have been identified in eukaryotes (www.bbcm.univ.trieste.it/tossi/pag5.htm). During recent years it has become increasingly evident that many AMPs, such as defensins and cathelicidins, are multifunctional, also mediating chemotaxis, apoptosis, and angiogenesis [4]–[6]. Conversely, molecules previously not considered as AMPs, including proinflammatory and chemotactic chemokines [7], neuropeptides [8], peptide hormones [9],[10], the anaphylatoxin peptide C3a [11],[12], growth factors [13] and kininogen-derived peptides [14]–[17] have recently been found to exert antibacterial activities.

Histidine-rich glycoprotein (HRG) is a plasma protein which was first isolated in 1972 by Heimburger *et al.* [18],[19]. The protein is present in human plasma at 1.5–2 µM, but the local concentration when HRG is released from activated platelets is likely to be higher [20]–[22]. It is a type 3 cystatin family protein [23], along with α-2-HS-glycoprotein/fetuin-A, fetuin-B and kininogen, and is found in vertebrates as well as in some invertebrates. The structure contains two cystatin-like domains, a central histidine-rich region (HRR) with highly conserved GHHPH tandem repeats flanked by proline-rich regions, and a C-terminal region [20]. This modular structure of HRG facilitates multiple interactions, involving ligands such as heparin, plasminogen, fibrinogen, thrombospondin, heme, IgG, FcγR, and C1q. Due to its high content of histidine residues (∼13%), which are concentrated to the HRR, HRG can acquire a positive net charge either by incorporation of Zn^2+^, or by protonation of histidine residues at acidic conditions [20]. In this context it has been proposed that HRG acts as a pH and Zn^2+^ sensor, providing a mechanism for regulating the various activities of HRG [24]. HRG has recently been ascribed antiangiogenic [25] effects *in vitro*, as well as antitumor [26] effects *in vivo*. Recent studies on Hrg^−/−^ mice furthermore suggest that HRG plays a role as both an anticoagulant and an antifibrinolytic modifier, and may regulate platelet function *in vivo* [22].

Previous work has also demonstrated that HRG exert direct antibacterial activities *in vitro* which are dependent on Zn^2+^and pH [27]. However, as many cationic proteins and peptide sequences display antimicrobial properties *in vitro*, the ultimate role(s) of HRG in innate immunity *in vivo* still remained unresolved. During the course of our studies, we observed that HRG had a significant activity against *Candida*. *Candida*, an eukaryote, is present as a commensal at mucosal surfaces and on skin. Although it may cause life-threatening sepsis in immunocompromised individuals it seldom causes invasive disease in immunologically normal individuals [28]. We therefore speculated that HRG could constitute a natural defence against *Candida* infections. In the present study we show, using a combination of microbiological, biochemical, and biophysical methods, that HRG exerts a potent antifungal activity particularly at low pH, which is mediated via its HRR, and targets ergosterol-rich membrane structures such as those of *Candida*. In mouse infection models, HRG protects against systemic infection by *Candida*, indicating a previously undisclosed antifungal role of HRG in innate immunity.

## Results

### Antifungal activity of HRG and binding to *Candida* cells

**Figure 1:**
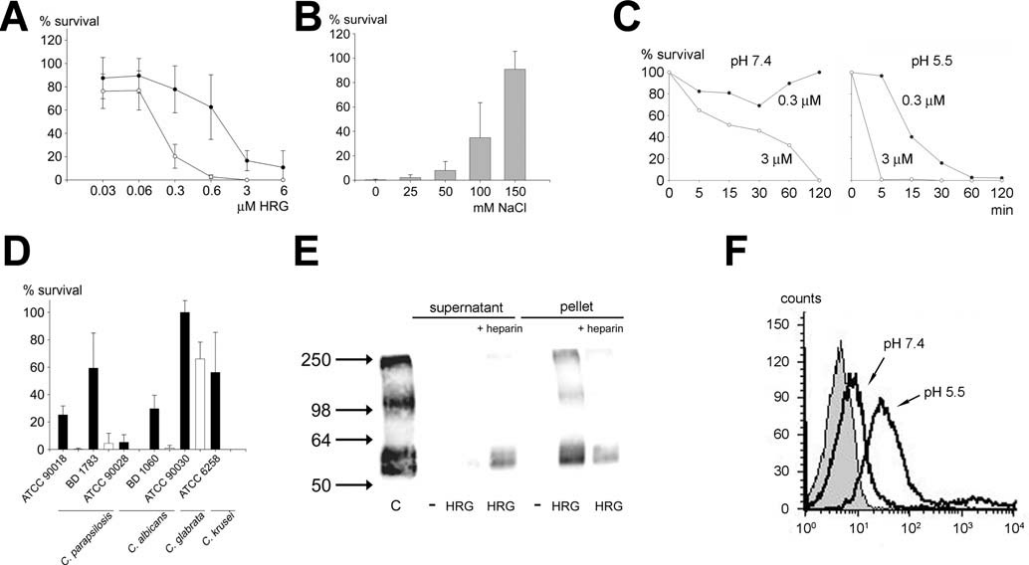
Antifungal activity and binding of HRG to *Candida*. (A) Antifungal activity of HRG. *C. parapsilosis* ATCC 90018 (1×10^5^ cfu) was incubated with purified human HRG at concentrations ranging from 0.03 to 6 µM for 2 hours in 10 mM Tris, pH 7.4 (•) or 10 mM MES, pH 5.5 (○), plated and the number of cfu determined (n = 6). (B) Antifungal effects of HRG in salt. *C. parapsilosis* ATCC 90018 (1×10^5^ cfu) was incubated with 6 µM HRG for 2 hours in 10 mM MES, pH 5.5 containing 0, 25, 50, 100 or 150 mM NaCl, plated and the number of cfu was determined. (C) Killing kinetics. 0.3 or 3 µM HRG were incubated with 1×10^5^ cfu *C. parapsilosis* ATCC 90018 for 0, 5, 15, 30, 60 or 120 minutes in 10 mM Tris, pH 7.4 (•) or 10 mM MES, pH 5.5 (○), plated and the number of cfu determined. (D) Antifungal activity of HRG against different strains of *Candida.* 3 µM HRG were incubated with 1×10^5^ cfu *C. parapsilosis* ATCC 90018 or BD 17837, *C. albicans* ATCC 90028 or BD 1060, *C. glabrata* ATCC 90030 or *C. krusei* ATCC 6258 in 10 mM Tris, pH 7.4 (black bars) or 10 mM MES, pH 5.5 (white bars) for 2 hours, plated and number of cfu determined (n = 6). (E) Binding of HRG to fungi. *C. parapsilosis* (1×10^5^ cfu) was incubated with HRG (0.6 µM) in 10 mM MES, pH 5.5. For inhibition studies, heparin (50 µg/ml) was added. Samples were centrifuged and the pellet and supernatants were extracted and run on 8% SDS-PAGE under reducing conditions. HRG was detected by western and immunoblotting using polyclonal antibodies against GHH20. Purified HRG was used as a positive control (labeled C). (F) Flow cytometry analysis of binding of HRG to fungal membranes. *C. parapsilosis* (5×10^7^ cfu) were incubated with FITC-labeled HRG in 10 mM Tris pH 7.4 or 10 mM MES pH 5.5.

In order to assess possible antifungal effects of HRG, we tested the activity of the protein against various *Candida* isolates. HRG was shown to be antifungal against *C. parapsilosis* at normal pH (10 mM Tris, pH 7.4), and the activity was significantly increased in low pH buffer (10 mM MES, pH 5.5) (Figure 1A). It is well-known that activities of AMPs and antimicrobial proteins are dependent of the microenvironment. For example, various chemokines, defensins, LL-37 as well as heparin binding protein are partly, or completely, antagonized by high salt conditions or the presence of plasma proteins *in vitro* [27],[29],[30]. Therefore, the influence of salt was tested. The results showed that HRG partially retained antifungal activity at physiological Cl^−^ levels (0.1 M) but only at low pH (Figure 1B). The antifungal activity against *C. parapsilosis* was both time- and dose-dependent (Figure 1C). In subsequent experiments various *Candida* strains (*C. parapsilosis*, *C. albicans*, *C. glabrata* and *C. krusei*) were incubated with HRG (at 3 µM) at neutral as well as low pH. Figure 1D demonstrates, in line with the above experiments, that HRG is particularly active at low pH. Thus, *C. parapsilosis*, *C. albicans* and *C. krusei* were all nearly completely killed by HRG at low pH, whereas *C. glabrata* exhibited a partial resistance at this concentration of HRG, the latter in analogy to *C. glabrata* displaying some resistance against histatin 5 [31]. Next, to investigate the binding of HRG to fungi, *C. papapsilosis* was incubated with HRG at low pH, washed, and analysed by immunoblotting. Since previous results indicated that the HRR of HRG, which binds heparin/heparan sulfate, mediates antibacterial effects [27], heparin was added for competition of binding to *Candida*. Figure 1E shows that HRG was able to bind to the fungal cells and that the binding was partially inhibited by an excess of heparin. This finding is compatible with the observation that heparin completely blocks the antifungal effect of HRG (Figure S1). As demonstrated by flow cytometry, HRG bound to *C. parapsilosis* at neutral pH, and the binding was significantly increased at pH 5.5 (Figure 1F), results compatible with the fungal killing assays (Figure 1A). In summary, therefore, the results demonstrate that the antifungal actions of HRG were pH-dependent and likely mediated via the heparin-binding region of the protein.

### Membrane-permeabilizing effects of HRG

**Figure 2:**
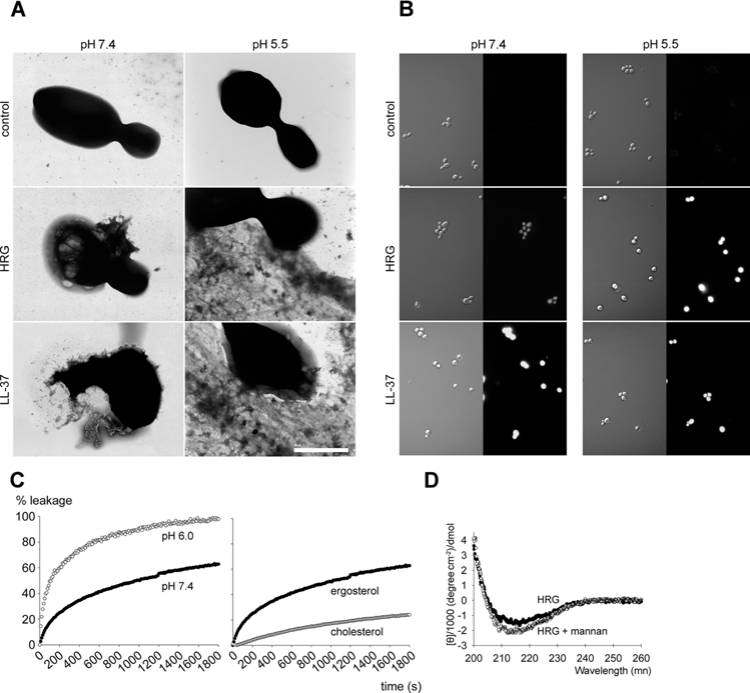
HRG induces membrane permeabilisation of *Candida* cells as well as liposomes. (A) Negative staining and electron microscopy analysis of *C. parapsilosis* exposed to HRG. *C. parapsilosis* ATCC 90018 were incubated in the absence of HRG in 10 mM Tris, pH 7.4 or 10 mM MES, pH 5.5. These fungi did not exhibit signs of membrane perturbations. In contrast, when treated with 10 µM HRG in 10 mM Tris, pH 7.4 or 10 mM MES, pH 5.5 membrane damage, blebbing and ejection of cytoplasmic components was observed. Fungi treated with 10 µM LL-37 was used as a positive control for membrane damage. The scale bar corresponds to 2 µm. (B) Fungal viability after incubation with HRG and LL-37. *C. albicans* ATCC 90028 were incubated with 10 µM HRG or LL-37 in either 10 mM Tris, pH 7.4 (left panel) or 10 mM MES, pH 5.5 (right panel). The left images in each row are Nomarski Differential Interference Contrast images, whereas the right images show FITC fluorescence of fungi. (C) Effects of 1 µM HRG on liposome permeability. Left panel. Increase in HRG-induced permeabilization of ergosterol-containing liposomes is detected at pH 6.0. Right panel. Increased HRG-induced lysis of (at pH 7.4) ergosterol containing liposomes. (D) CD spectroscopy of HRG under different conditions. CD spectra for 0.25 µM HRG in buffer and in presence of *S. cerevisiae* mannan are presented.

Many AMPs kill microbes by membrane lysis, while others may translocate through membranes and subsequently interact with intracellular targets, such as DNA and mitochondria, all eventually resulting in microbial killing [32],[33]. Considering the antifungal effects and the binding to *Candida* cells, it was of interest to further study the possible mode of action for HRG on *Candida*. Electron microscopy demonstrated that HRG caused membrane breaks in *Candida* cells and release of cytoplasmic components (Figure 2A), effects particularly noted at low pH, where significant extracellular material was detected. The effects were similar to those observed after treatment with the “classical” human AMP LL-37 (Figure 2A). These data suggest that HRG acts on fungal membranes, however they do not demonstrate the exact mechanistic events, as secondary metabolic effects on fungi also may trigger death and membrane destabilization. Therefore, the impermeant dye FITC was used to assess permeabilisation. The results showed that HRG indeed was able to permeabilise *Candida* membranes (Figure 2B). In line with previous antifungal and binding experiments (see Figure 1A and 1F), the permeabilisation was most apparent at low pH.

These results were further substantiated by the use of a liposome model to assess membrane permeabilisation. In correspondence with the effects of HRG on *Candida*, HRG caused liposome leakage. Compatible with the pH sensitivity observed for HRG, the molecule preferably disrupted ergosterol-containing liposomes at pH 6.0 when compared with pH 7.4 (Figure 2C, left panel). Notably, ergosterol-containing liposomes, mimicking fungal membranes, were more sensitive than cholesterol-containing ones, mimicking mammalian membranes (Figure 2C, right panel). These results are in agreement with numerous previous findings on the membrane-stabilizing effects of cholesterol [34], as well as the findings that ergosterol induce less membrane stability in phospholipids than cholesterol [35]. At lower pH, protonation of histidine groups (pKa for the isolated histidine group is approximately 6.5), effectively increases the net charge density of HRG, thus the observed effects are compatible with findings previously reported for histidine-containing consensus peptides and histidine-rich endogenous peptides [36],[37]. Also noteworthy is that HRG did not display any major conformational changes either at low pH, or in the presence of fungal mannan (Figure 2D) or ergosterol-containing phospholipid liposomes (not shown). Hence, large-scale conformational changes appear not to be critical for the antifungal action of HRG. Taken together, the combination of electron microscopy, FITC-studies, and liposome data demonstrates that HRG acts at least in part through membrane disruption, although it is possible that additional intracellular effects of HRG may also contribute to fungal death. It is also notable that the observed effects were most marked and consistent at low pH. At neutral pH, binding (Figure 1B), as well as permeabilization (Figure 2A and 2B) was less apparent and these observations reflected the diminished antifungal effects at pH 7.4 (Figure 1A and 1D).

### Antifungal regions of HRG

**Figure 3:**
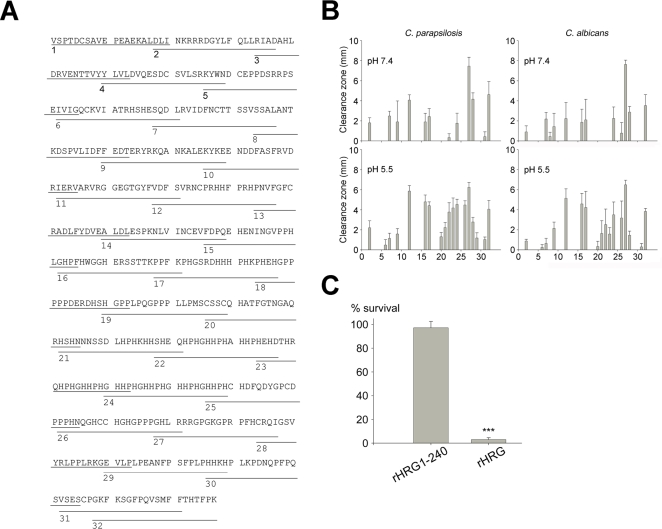
The antifungal activity of the histidine-rich domain of HRG is significantly increased at low pH. (A) Sequence of HRG and synthetic peptides used in this study are indicated. (B) Screening of antifungal epitopes of HRG. 20-mer peptides spanning the whole sequence of HRG (for sequences see Table S1) were used in radial diffusion assays against *C. parapsilosis* ATCC 90018 and *C. albicans* ATCC 90028 in 10 mM Tris, pH 7.4 or in 10 mM MES, pH 5.5. A 4 mm diameter well was loaded with 6 µl of 100 µM peptide. The clearance zones (mm) were measured after an overnight incubation at 27°C (n = 6). (C) Comparison of the antifungal activity of rHRG and the truncated version rHRG1-240. *C. parapsilosis* ATCC 90018 (1×10^5^ cfu) was incubated with 0.6 µM rHRG or rHRG1-240 in 10 mM Tris, pH 7.4 or in 10 mM MES, pH 5.5. Samples were plated and the number of cfu was determined. Significance was determined using Kruskall-Wallis one-way ANOVA analysis (*** p<0.001, n = 6).

In order to explore the structure-function relationships of epitopes of HRG, overlapping peptide sequences comprising 20mers (Figure 3A and Table S1) were synthesized and screened, at both neutral and acidic pH, for antifungal activities against *C. parapsilosis* as well as *C. albicans*. The experiments identified several antifungal regions. In particular peptides no. 20–24 and 26, spanning the HRR, displayed a significant antifungal activity against both *Candida* strains at low pH (Figure 3B). There was a clear correlation with net charge (at the respective pH) of the various peptide regions and their observed antifungal activity (Figure S2). Although intuitively apparent (Figure 3B), the analysis furthermore showed that peptides derived from the HRR were (with the exception of the K and R-rich peptide no. 27) characterized by an increase in net charge at low pH (Table S1 and Figure S2).

In order to further study the importance of the HRR we investigated the activity of recombinant HRG (rHRG) and a truncated version (rHRG1-240), lacking the HRR and C-terminal domain. In contrast to full-length rHRG, truncated rHRG (0.6 µM) displayed no activity at pH 5.5 against *Candida* (Figure 3C). Taken together, considering the well-known heparin binding capacity of HRR, its pH dependence, as well as the absence of antifungal activity of rHRG1-240, it was logical to focus on the HRR of HRG in the subsequent studies of antifungal activity.

**Figure 4:**
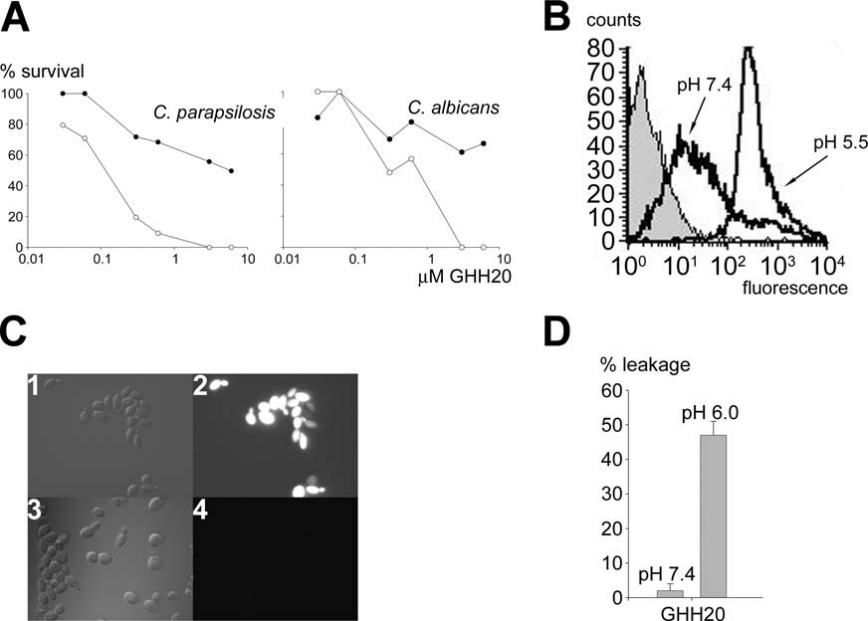
Antifungal activity and fungal binding of GHH20 peptide. (A) Antifungal activity of GHH20. *C. parapsilosis* ATCC 90018 *or C. albicans* ATCC 90028 (1×10^5^ cfu) were incubated with GHH20 peptide (0.03 to 6 µM) for 2 hours in 10 mM Tris, pH 7.4 (•) or 10 mM MES, pH 5.5 (○), plated and the number of cfu determined. A representative experiment (of three) is shown. (B) Flow cytometry analysis of binding of GHH20 to fungal membranes. *C. parapsilosis* (5×10^7^ cfu) were incubated with 20 µg TAMRA-labeled GHH20 in 10 mM Tris pH 7.4 or 10 mM MES pH 5.5. (C) Binding of 2 µg TAMRA-labeled GHH20 peptide to *C. parapsilosis* ATCC 90018 and inhibition by an excess of heparin. *C. parapsilosis* were incubated with TAMRA-labeled GHH20 in 10 mM MES (panel 3), pH 5.5 or the same buffer supplemented with heparin (50 µg/ml) (panel 4). The left panel shows Nomarski images (1 and 3), whereas the right panel shows red fluorescence of peptide bound to fungi. (D) The GHH20 peptide permeabilizes ergosterol-containing liposomes preferably at pH 6.0. 1 µM GHH20 was used (n = 6).

The HRR contains 12 tandem repeats of five consensus sequences of amino acids, GHHPH [20], a motif highly conserved among various vertebrate species [27]. To examine the activity of this sequence motif further, a 20-mer peptide (GHHPH)_4_ [16],[27] was chosen for further studies. Similar to intact HRG, GHH20 was antifungal against *C. parapsilosis* and *C. albicans*, particularly at low pH (Figure 4A). As demonstrated by FACS analysis, Tetramethyl-6-Carboxyrhodamine (TAMRA)-labeled GHH20 peptide bound to *C. parapsilosis*, and in correspondence with the antifungal data, the binding was stronger at pH 5.5 when compared to neutral pH (Figure 4B). As illustrated by fluorescence microscopy, TAMRA-labeled GHH20 showed a significant binding to *Candida* at pH 5.5 (Figure 4C). As with the HRG holoprotein, heparin abolished the binding, reflecting the heparin-binding capacity of this region of the HRR [27]. Also in line with the above experiments on fungi, GHH20 preferably disrupted liposomes at pH 6.0, with no significant activity at pH 7.4 (Figure 4D). The GHH20 peptide caused liposome leakage within a few hundred seconds (not shown), which contrasted to the significantly slower HRG-induced liposome leakage (Figure 2B), likely a manifestation of the much higher molecular weight of the holoprotein. Again as with intact HRG, CD spectroscopy showed that GHH20 displayed no major conformational changes associated with the histidine protonation at low pH, nor on interaction with phospholipid liposomes or mannan (not shown). Taken together, the GHH20 peptide showed similar characteristics as the holoprotein HRG with respect to activity, binding, and membrane permeabilisation.

### HRG is found in biological fluids and is active in plasma and in fibrin clots

**Figure 5:**
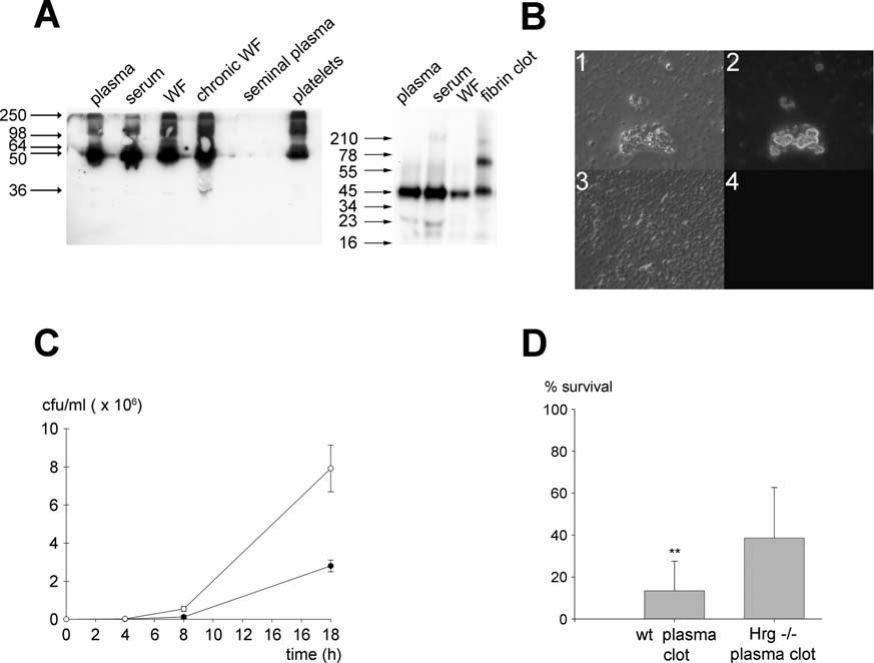
Localization and activities of HRG. (A) Analysis of HRG in biological fluids. The indicated biological materials were electrophoresed on a 8% gel (Tris-Glycine, non-reducing conditions) (left panel) or on a 16.5% Tris-Tricine gel under reducing conditions (right panel) and transferred to a nitrocellulose membrane. Western blot was performed using polyclonal antibodies directed against the GHH20 epitope of HRG. (B) Localization of HRG in fibrin clots. Human control plasma (panel 4) or plasma depleted of HRG (panel 2) were incubated with FITC-labeled HRG and clots were generated overnight after addition of 10 mM Ca^2+^ at 37°C. The clots were mounted on slides and visualized by fluorescence microscopy. The left side shows Nomarski images (1 and 3), whereas the right part shows fluorescence of HRG associated with the clots. (C) Candida growth in plasma. *C. parapsilosis* (2×10^7^ cfu) was inoculated in human plasma (•) or human HRG-deficient plasma (○) and incubated at 27°C for 0, 4 ,8 or 18 hours and the number of cfu was determined (n = 6). (D) Antifungal activity of HRG *ex vivo.* Mouse control plasma or plasma of Hrg^−/−^ mice were used to form fibrin clots in the presence of 10 mM Ca^2+^. Clots were incubated with *C. parapsilosis* ATCC 90018 (1×10^5^ cfu) in 10 mM MES, pH 5.5 for 2 hours, plated and the number of cfu determined (p = 0.043, n = 6).

In order to investigate the functional relevance of the above *in vitro* activities, we first tested the role of HRG against fungi in relevant physiological “settings” *ex vivo*. Initial results showed that HRG was detected in blood fractions (plasma, serum) and in platelets, also in wound fluid from acute wounds, and chronic leg ulcers (Figure 5A). The latter wound type is characterized by unregulated and excessive proteinase activity leading to degradation of many plasma proteins [38],[39]. However, compared with plasma and serum HRG, the molecule was not fragmented in this chronic wound fluid fraction (Figure 5A). The protein was also detected in fibrin clots (Figure 5A) but not present in seminal plasma. It is of note that the molecule migrated aberrantly in the used gel systems; relative 55–60 kDa in 8% gels (Tris-Glycine) and 45–50 kDa in 16.5 gels (Tris-Tricine). Identical serum and plasma preparations of HRG were used in the two gel systems, and recombinant HRG showed the same anomalous migration (not shown). In addition to its presence in plasma and other biological fluids, HRG occurs at significant levels in, and binds avidly to, fibrin clots [40]. Coagulation was initiated in normal and HRG-deficient human plasma in the presence of FITC-labeled HRG (Figure 5B). FITC-labeled HRG bound to clots derived from HRG-deficient plasma, and notably, it appeared to be present at clot boundaries, suggesting that it may “coat” the clot surfaces. In clots from normal plasma, no staining was seen, indicative of an inhibition of binding of FITC-HRG by the excess of endogenous HRG (∼150 µg/ml). Clots, physiologically important “barriers”, formed during hemostasis and infection, could thus constitute a unique milieu with high levels of surface-immobilized HRG. Considering the above results we investigated whether the presence of HRG could reduce the growth of *Candida* in plasma. Firstly, the growth of *C. parapsilosis* was investigated in normal human plasma and in plasma depleted of HRG. The results showed that *C. parapsilosis* multiplied significantly faster in HRG-depleted human plasma (Figure 5C). Analogous results on fungal growth were observed using plasma from mice deficient in HRG (data not shown). It is of note that these results do not exclude the possibility that other antifungal mechanisms may be involved, such as those dependent of complement activation. Furthermore, although the total protein levels (as determined by the Bradford method) and contents (as assessed by SDS-PAGE on 8% gels, not shown) were the same in depleted plasma (51.0+/−1.2 g/l) when compared with control plasma (51.7+/−3.3 g/l), it cannot be excluded that additional changes of low abundance proteins, induced by passage over Ni-NTA agarose could affect *Candida* growth. Nevertheless, the observation that similar results were obtained with the mice plasmas points at HRG as the main factor responsible for the partial growth inhibition noted. Furthermore, as demonstrated in Figure 5D, fibrin clots derived from plasma of HRG deficient mice were significantly more prone to infection by *C. parapsilosis* than clots from wild-type mice, and similar results were obtained with human plasma depleted of HRG when compared with normal plasma (not shown). The observation that clots devoid of HRG showed detectable, although reduced, antifungal activity (Figure 5D) suggest the existence of other yet unidentified factors in clots also mediating fungal killing. Nevertheless, the results indicate that HRG contributes to antifungal activity under physiological conditions.

### *In vivo* role of HRG

**Figure 6:**
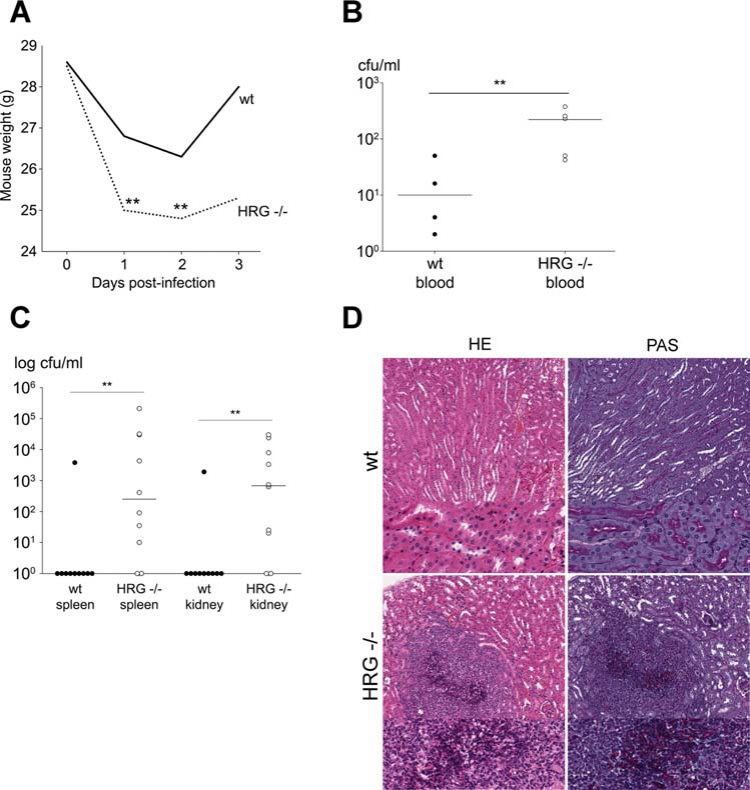
Candida produces severe infection in Hrg^−/−^ mice. (A) Weight loss in mice after infection with *C.albicans*. C57BL/6 (solid line) and C57BL/6 Hrg^−/−^ (dotted line) mice were infected (i.p.) with 1×10^9^ cfu of *C. albicans*, and the body weight was followed from day 0 to day 3 (n = 6,4 and 2, respectively) (p = 0.02). (B) Fungal dissemination to the bloodstream. C57BL/6 (•) (n = 4) and C57BL/6 Hrg^−/−^ (○) (n = 5) mice were infected as above and the animals were sacrificed on day 2 and number of cfu in blood was determined (p = 0.032). (C) HRG suppresses fungal dissemination to the spleen and kidney. C57BL/6 (•) and C57BL/6 Hrg^−/−^ (○) mice were infected as above and the cfu of *C. parapsilosis* in spleen and kidney was determined (p = 0.009, n = 10) (D) In two animals kidneys were collected on day 3, fixed, sectioned and then stained with hematoxylin and eosin (HE) (left panel) or PAS (right panel) (upper section; magnification ×10 and lower section; magnification ×30).

To investigate the role of HRG during *Candida* infection *in vivo*, we designed a mouse model of intraperitoneal infection with *C. albicans*. After infection, the body weight of the mice was followed for three days (Figure 6A). Hrg^−/−^ mice showed a significantly increased weight loss at day 1 and 2 (p = 0.02) when compared with wild type mice, and the wild type mice regained their initial weight after three days. Blood samples were collected from the animals 2 days post infection, and the fungal load in blood was determined (Figure 6B). A significantly higher amount of *Candida* cells was detected in the blood of Hrg^−/−^ mice when compared with wild type mice (p = 0.032), indicating that a systemic infection has developed in HRG-deficient mice. In a similar experiment, we determined the ability of the fungi to establish infection in target organs distant from the site of administration. The spleen and kidney were harvested 3 days after initiation of intraperitoneal infection and the fungal load was determined. The results showed significant differences between Hrg^−/−^ mice and the wild type mice; one animal out of 10 in the control group showed fungal load in the spleens and kidneys compared with 8 out of 10 in the Hrg^−/−^ group (p = 0.009) (Figure 6C). Histopathological examination of the kidney tissues from Hrg^−/−^ mice showed dense neutrophil infiltrates and notably, *Candida* cells were visualised by PAS staining in the centre of these infiltrates (Figure 6D). These results show a striking protective role for HRG against invasive *Candida* infection *in vivo*.

## Discussion

The key findings in our study are the identification of an antifungal activity of HRG *in vivo* together with the characterization of possible epitopes of HRG mediating this effect, as well as mechanistic data on HRG targeting of *Candida* membranes. The results have implications for our understanding of novel antifungal properties of HRG, and demonstrate that HRG constitutes a previously undisclosed natural and antimicrobial defence system.

From a structural perspective, several lines of evidence indicate that the HRR is, at least to a significant extent, responsible for the HRG interaction with *Candida* membranes. Although the 3D structure of HRG has not yet been determined, modelling studies suggest that the HRR of HRG forms a polyproline (II) helical structure with numerous histidines. At physiological pH, HRG is net negatively charged (pI 6.45). However, due to its high content of histidine residues (∼13%), which are concentrated to the HRR, it can acquire a positive charge by protonation [20],[41], and this in turn likely facilitates the interactions between HRG and *Candida*. These results were substantiated by the finding that a region of HRG containing the motif sequence GHHPH, was antifungal, and that low pH enhanced this activity. The high conservation of this sequence among vertebrates likely reflects its importance for membrane interactions of HRG [27]. However, as evident in Figure 3B, there are also other antifungal regions in the protein, active irrespective of pH in the interval investigated, an observation compatible with the antifungal activity of HRG detected at neutral pH. It should be pointed out however, that the peptide data do not reflect the complex structure-activity relationships of the holoprotein. Although the CD experiments did not detect any major conformational changes upon interaction with liposomes or polysaccharides, it cannot be ruled out that conformational changes mediated by HRR interactions with intact fungal cells lead to the exposure of additional antimicrobial epitopes in the molecule. Nevertheless, a recombinant and truncated variant of HRG, lacking the histidine-rich and C-terminal domains, was not active against *Candida*, pointing to the HRR as an important, possibly the most important, effector of HRGs antifungal effects.

Many histidine-rich AMPs are known, among these the clavanins [36], histatins, and calprotectin [42]. We have previously shown that the antibacterial effects *in vitro* of various histidine-rich peptides, both consensus motifs and peptides derived from domain 5 of HMW kininogen [17] and from HRG [27] are enhanced at low pH or upon addition of Zn^2+^. Others have reported that the antimicrobial activity of clavanins were substantially increased in low pH as compared with neutral pH [36]. Furthermore, the antimicrobial effect of histatin 5 is enhanced at low pH [43], and histidine-rich variants of magainin, the LAH4-peptides, were recently shown to have increased antibacterial activity in low pH compared to neutral pH [37]. Taken together, the pH dependent activity of HRG is thus comparable to other histidine-rich proteins and peptides, and provides an additional link between pH sensitive AMPs and HRG. However, contrasting to histatins, which translocate through *Candida* membranes, bind mitochondria, and induce cell death by non-lytic ATP-release [44], HRG acts directly on fungal membranes.

Many AMPs are generated by proteolysis of larger, and non-antimicrobial holoproteins. For example, the cathelicidin LL-37 is released from hCAP18, and other AMPs are proteolytically generated from complement factor C3 and high molecular weight kininogen [3], [11], [12], [14]–[17]. Considering that intact HRG is antifungal, proteolysis of this molecule does not appear to be needed for activity. It is of note that like HRG, several antimicrobial proteins are antimicrobial *per se*, including bacterial permeability increasing protein, serprocodins such as proteinase 3, elastase and heparin binding protein, as well as lactoferrin [27],[45]. However, it is also described that antibacterial proteins, such as bacterial permeability increasing protein and lactoferrin, may give rise to peptides exerting antibacterial activities [46],[47]. Likewise, it has been shown that HRG may be degraded by plasmin [48], as well as in patients undergoing thrombolytic therapy [49] and bioactive fragments of HRG are involved in antiangiogenesis [26],[41]. Thus, although a major fragmentation of HRG was not observed in this work, e.g., in wound fluid and after binding to fibrin, it is likely that degradation of HRG may occur at sites of high proteolysis and plasmin activity. Indeed, the finding that the HRG-derived peptide GHH20, as well as numerous other other 20mer peptides were antifungal, and as particularly noted for HRR-derived peptides, exhibiting a similar pH dependence as HRG, exemplifies that the holoprotein is not a prerequisite for antifungal action. Clearly, such possibilities need to be addressed in future studies.

As previously mentioned, HRG is involved in various aspects of angiogenesis, coagulation, and fibrinolysis [20], reflecting its interactions with ligands such as heparin, plasminogen, fibrinogen, and thrombospondin. Additionally, it acts as an opsonin by bridging FcyRI receptors on macrophages to DNA on apoptotic cells, stimulating phagocytosis [50], and modulates the binding of IgG and immune complexes to FcγRI [50]. Considering these multiple roles, it is likely that HRG binding to microbial surfaces could induce additional “down-stream” effects, such as modulation of plasminogen activity and phagocytosis. The history of “classic” AMPs have shown that these molecules, initially believed to take part merely in direct microbial killing, have extended their roles into the ability to act as chemokines and to induce chemokine production leading to recruitment of leukocytes, promotion of wound healing, and an ability to modulate adaptive immunity [51]. Indeed, as interest in the *in vivo* functions of host defence peptides is increasing, it is important to consider the direct antimicrobial and immunomodulatory properties observed. Nevertheless, several findings in this study unequivocally demonstrate that HRG, like many AMPs, acts directly on microbes. Thus, in addition to the antifungal *in vitro* data, the enhanced fungal growth in HRG-deficient plasma, as well as the finding that *Candida* was detected at higher levels in blood of Hrg^−/−^ animals, indicates a direct antifungal action of the molecule. It is also interesting to note that these HRG deficient animals have also been shown to be more susceptible to *Streptococcus pyogenes* infection (Shannon et al, unpublished results). However, considering both AMP and HRG multifunctionality *in vitro* as well as *in vivo*, it may be envisaged that additional actions, resulting in the observed antifungal effects, will likely be revealed. All of these effects may be dependent on binding of HRG to microbes and subsequent interactions with cells (e.g., neutrophils and macrophages) in different compartments (e.g., skin, internal organs, and blood). In this respect, the pH dependence of HRG is particularly interesting and relevant. It is well known that infection foci, including abscesses, are characterized by low pH levels reaching as low as pH 5, due to increased anaerobic metabolism and lactate production, as well as leukocyte mediated oxidative burst and subsequent acidification [52]. The capacity of HRG to kill *Candida* at these pH levels and the corresponding increase in salt-resistance at low pH suggest that HRG could target infection foci, resulting in a physiologically relevant concentration and localization of antifungal activity. As previously mentioned, HRG's opsonising activity could hypothetically lead to enhanced phagocytosis. Although it remains to be investigated, such localisation of antifungal activity to endosomal compartments, where acidification could result in enhanced HRG-mediated killing of phagocytosed fungi, could serve as an effective way of eliminating invading *Candida* cells at sites of tissue inflammation without releasing potentially toxic microbial components.

Again hypothetically, genetic deficiencies of HRG or acquired functional defects could provide interesting clues with respect to functional roles of HRG. In some patients, reduced levels of HRG are associated with a thrombophilic phenotype, indeed compatible with the phenotype observed in Hrg^−/−^ mice, which had a shorter prothrombin time [22]. As these patients still have ∼20–50% of normal levels of HRG, the human phenotype of complete absence of HRG remains, however unknown. Although patients with low levels of HRG have not been reported to be more prone to infections, it must be remembered that examples from deficiencies of particular innate immune proteins, e.g., complement and mannose-binding lectin, illustrate that even homozygous deficiency and a complete absence of a particular innate immune molecule may give rise to surprisingly mild symptoms. For example, patients with mannose-binding lectin deficiencies are normally not at risk of developing infections unless compromised by immune suppression or severe disease [53]. In this context, it is particularly interesting that antibodies against HRG have been detected in patients with antiphospholipid syndrome [54], a disease associated with thrombodiathesis and systemic lupus erythematosus. Notably, the latter disease is associated with an increased risk for opportunistic infections, including *Candida* [55]. Taken together, and considering the role of HRG in innate immunity, it should be of interest to study potential associations between functional inactivation(s) or deficiencies of HRG as well as genetically determined differences, in relation to the occurrence of infections.

During the last three decades, research on innate immune molecules has demonstrated the significance of the innate immune system for prevention of invasion by microbes at biological boundaries. Previous studies have emphasized that various molecules, such as “classic” AMPs, complement factors, and cytokines, bridge between innate and adaptive immunity. The present work adds another significant component to this family of molecules, the plasma protein HRG.

## Materials and Methods

### Materials

The peptides GHH20 (GHHPHGHHPHGHHPHGHHPH) and histatin 5 (DSHAKRHHGYKRKFHEKHHSHRGPY) were synthesized by Innovagen AB (Lund, Sweden), and were of >95% purity. The purity and molecular weight was confirmed by MALDI-TOF MS analysis (Voyager, Applied Biosystems). 20-mer synthetic peptides (PEP-screen) spanning the sequence of HRG (Table 1) were obtained from Sigma-Genosys (St Louis, MO). Polyclonal rabbit antibodies against GHH20 and TAMRA-labeled GHH20 were from Innovagen AB (Lund, Sweden). HRG was FITC-labeled using the FluoroTag FITC Conjugation Kit (Sigma, St Louis, MO). Human serum and plasma were collected from healthy volunteers. Sterile wound fluids were obtained from surgical drainages after mastectomy. The use of human wound fluid was approved by the Ethics Committee at Lund University (LU 708-01). Seminal plasma was collected at the Fertility Center at Malmö University Hospital, Sweden. The GenBank (http://www.ncbi.nlm.nih.gov/Genbank/index.html) accession number of human histidine-rich glycoprotein is NP_000403.

### Fungal strains

The fungi *Candida parapsilosis* BD 17837 and *Candida albicans* BD 1060 were clinical isolates. *C. parapsilosis* ATCC 90018, *C. albicans* ATCC 90028, *Candida glabrata* ATCC 90030, and *Candida krusei* ATCC 6258 isolates were from the American Type Culture Collection (ATCC, Rockville, MD).

### Purification of human HRG

Serum HRG was purified using nickel-nitrilotriacetic acid (Ni-NTA) agarose as described before [27]. The concentration of the protein was determined using the Bradford method [56].

### Production and purification of recombinant HRG (rHRG and rHRG1-240)

Recombinant His-tagged HRGP and truncated version of HRG (HRG1-240), containing amino acids 1-240 was produced and purified as previously described [26],[27].

### Western blot

Plasma, serum, wound fluids, seminal plasma (1 µl), and platelets (fluid from 1×10^3^ cells, disrupted by freeze thawing) were electrophoresed on 8% SDS-polyacrylamide (SDS-PAGE) gel or an 16.5% Tris-tricine gel and transferred to a nitrocellulose membrane (Hybond-C, GE Healthcare BioSciences, Little Chalfont, UK) [57]. The membrane was incubated in 3% skimmed milk in 10 mM Tris, 0.15 M NaCl, pH 7.4 for 1 h at room temperature, followed by incubation for 1 h with rabbit polyclonal antibodies against GHH20 (diluted 1:1000 in the same buffer). The membrane was washed 3 times, and incubated again for 1 h with horseradish peroxidase-conjugated secondary swine anti rabbit antibodies diluted 1:1000 (Dako, Carpinteria, CA). The image was developed using the ECL system (Amersham Biosciences).

### Preparation of fibrin clots

Human plasma was subjected to a Ni-NTA agarose gel. The eluent (plasma completely depleted of HRG) was collected and used to form clots. Hrg^−/−^ and C57BL/6 (wild type) mice [22] were used for preparation of fibrin clots from plasma of the respective animals. Plasma deficient of HRG and normal plasma were incubated with a total concentration of 10 mM Ca^2+^ in eppendorf tubes at 37°C over night. Clots were washed three times and then stored in 10 mM 2-Morpholinoethanesulfonic acid (MES), pH 5.5. Clots (∼0.04g) were used in viable count experiments. To investigate the localization of HRG in fibrin clots, human plasma and HRG-deficient plasma were incubated with 10 µl FITC-labeled HRG (0.4 mg/ml) and then processed as before in the presence of 10 mM Ca^2+^ over night. The clots were then washed in distilled water and mounted on slides using Dako mounting media (Dako).

### Viable count assay

*C. parapsilosis*, *C. albicans*, *C. glabrata and C. krusei* were grown to mid-logarithmic phase in Todd-Hewitt (TH) medium (Becton and Dickinson, Maryland, USA) at 27°C and washed in 10 mM Tris, pH 7.4 or 10 mM MES, pH 5.5. For dose-response experiments, purified HRG or GHH20 (0.03–6 µM) were incubated with 1×10^5^ *C. parapsilosis* ATCC 90018 *or C. albicans* ATCC 90028 for 2 h at 37°C in 10 mM, Tris, pH 7.4 or in 10 mM MES-buffer, pH 5.5, plated on Sabouraud dextrose broth (Becton and Dickinson) agar, and incubated 48 hours at 27°C, whereafter the number of cfu was determined. In order to investigate the antifungal activity of HRG in presence of salt, 6 µM HRG were incubated with 1×10^5^ *C. parapsilosis* ATCC 90018 for 2 h at 37°C in 10 mM, MES, pH 5.5 containing 0, 25, 50, 100 or 150 mM NaCl, plated and the number of cfu was determined. In kinetic experiments, 0.3 and 3 µM HRG were incubated with *C. parapsilosis* ATCC 90018 for 5, 15, 30, 60, or 120 minutes in 10 mM MES, pH 5.5, plated and the number of cfu was determined. For determination of the effect of HRG on various *Candida* strains, HRG (3 µM) was incubated with *C. parapsilosis* ATCC 90018 or BD 17837, *C. albicans* ATCC 90028 or BD 1060, *C. glabrata* ATCC 90030 or *C. krusei* ATCC 6258 in 10 mM Tris, pH 7.4 or 10 mM MES, pH 5.5, plated and number of cfu determined. Truncated and full length recombinant HRG, 0.6 µM rHRG, or rHRG1-240 were incubated with *C. parapsilosis* (1×10^5^) for two hours and then plated and number of cfu determined. To investigate the *in vitro* antifungal activity of HRG, normal or HRG-deficient fibrin clots (∼0.04g) were incubated with *C. parapsilosis* ATCC 90018 for 2 h in 10 mM MES, pH 5.5, plated and number of cfu were determined. For inhibition studies, 0.3 µM HRGP were incubated with *C. parapsilosis* (1×10^5^) in 10 mM MES, pH 5.5, in presence or absence of heparin (50 µg) for two hours and then plated and number of cfu was determined. In all experiments, 100% survival was defined as total survival of fungi in the same buffer and under the same conditions in absence of peptide, protein, or clots. The p-values were determined using Kruskall-Wallis one-way ANOVA analysis.

### Radial diffusion assay

Radial diffusion assay (RDA) was performed essentially as described earlier [58]. *C. parapsilosis* ATCC 90018 and *C. albicans* ATCC 90028 were grown to midlogarithmic phase in TH-medium, and then washed with distilled water. 4×10^6^ colony forming units was added to 5 ml of the underlay agarose gel (0.03% (w/v) trypticase soy broth (TSB), 1% (w/v) low electroendosmosis type agarose (Sigma), 0.02% (v/v) Tween 20 (Sigma). The buffers used in the underlay gels were 10 mM Tris, pH 7.4 or 10 mM MES, pH 5.5. The underlay gel was poured into an 85-mm Petri dish. After agarose solidification, wells of 4 mm in diameter were punched, and 6 µl of peptide solution was added to each well. Buffers were used as a negative control. Plates were incubated at 28°C for 3 h to allow diffusion of the peptides. The underlay gel was then covered with 5 ml of molten overlay. Antimicrobial activity of a peptide is visualized as a zone of clearance around each well after 18–24 h of incubation at 28°C. Peptides were tested in concentrations of 100 µM.

### Binding of HRG to *Candida*

*C. parapsilosis* (1×10^5^ cfu) were incubated with 0.6 µM HRG in 50 µl 10 mM MES, pH 5.5, with or without heparin (50 µg/ml) for 2 h at 37°C, centrifuged and the pellet was washed three times in 10 mM MES, pH 5.5. The pellet and the supernatant were resuspended in SDS sample buffer, electrophoresed (8% SDS-PAGE), and then transferred to a nitrocellulose membrane. Western blotting was performed as above.

### Fluorescence microscopy

*C. parapsilosis* ATCC 90018 fungi were grown in TH medium at 27°C to mid-logarithmic phase. The fungi were washed in 10 mM Tris, pH 7.4, and resuspended in the same buffer. *C. parapsilosis* (2×10^6^/ ml) were incubated with 1 µl of TAMRA-labeled GHH20 (2 mg/ml) in 10 mM MES, pH 5.5, with or without heparin (50 µg/ml), left standing for 5 minutes on ice, and then washed twice in 10 mM Tris, pH 7.4. Fungi were fixed with 4% paraformaldehyde by incubation on ice for 15 minutes and in room temperature for 45 minutes. The fungi were then applied onto Poly-L-lysine coated cover glass and after an incubation time of 30 minutes, finally mounted on slides using Dako mounting media (Dako, Carpinteria, CA). In order to assess permeabilisation, *C. albicans* ATCC 90028 (2×10^6^ cfu) were incubated with HRG or LL-37 (both at 10 µM) in 10 mM Tris, pH 7.4 or 10 mM MES, pH 5.5 for 30 minutes at 37°C. Samples were transferred to Poly-L-lysine coated cover glass and incubated for 45 minutes at 37°C, washed and 2 µg of FITC were added in a volume of 200 µl, and incubated for 30 minutes at 30°C, washed and then fixed as above. Samples were visualized using a Nikon Eclipse TE300 inverted fluorescence microscope equipped with a Hamamatsu C4742-95 cooled CCD camera, a Plan Apochromat 100X objective and a high N.A. oil condenser.

### Negative staining and transmission electron microscopy

*C. parapsilosis* ATCC 90018 were grown in TH medium at 37°C to mid-logarithmic phase. The fungi were washed in 10 mM Tris, pH 7.4 or 10 mM MES, pH 5.5, and resuspended in the same buffer. HRG or LL-37 (10 µM) was incubated with *C. parapsilosis* (20×10^6^ cfu) for two hours in a total volume of 10 µl in Tris buffer, pH 7.4 or in MES buffer, pH 5.5. Samples of *C. parapsilosis* fungi suspensions were adsorbed onto carbon-coated copper grids for 1 min, washed briefly on two drops of water, and negatively stained on two drops of 0.75 % uranyl formate. The grids were rendered hydrophilic by glow discharge at low pressure in air. Specimens were observed in a Jeol JEM 1230 electron microscope operated at 60 kV accelerating voltage. Images were recorded with a Gatan Multiscan 791 CCD camera.

### Flow cytometry

*C. parapsilosis* ATCC 90018 were grown in TH medium at 27°C to mid-logarithmic phase. The fungi were washed in 10 mM Tris, pH 7.4 or 10 mM MES, pH 5.5 and resuspended in the same buffer. *C. parapsilosis* (5×10^7^ in a total volume of 0.5 ml) were incubated with 10 µl of FITC-labeled HRG (0.4 mg/ml) or 10 µl TAMRA-labeled GHH20 (2 mg/ml) in 10 mM Tris, pH 7.4 or in 10 mM MES, pH 5.5, let stand for 5 minutes on ice and then washed in 10 mM Tris, pH 7.4. The cells were fixed with 4% paraformaldehyde by incubation on ice for 15 minutes and in room temperature for 45 minutes. Flow cytometry analysis was performed using a FACS-Calibur flow cytometry equipped with a 15 mW argon laser turned a 488 mm (Becton-Dickinson, Franklin Lakes, NJ). The fungal population was selected by gating with appropriate settings of forward scatter (FSC) and sideward scatter (SSC). The FL1 fluorescence channel (λ_em_ = 530 nm) was used to record the emitted fluorescence of FITC, and the FL3 fluorescence channel (λ_em_ = 585 nm) was used to record the emitted fluorescence of Texas red.

### Liposome preparation and leakage assay

Dry lipid films were prepared by dissolving dioleoylphosphatidylcholine (1,2-dioleoyl-sn-Glycero-3-phoshocholine, >99% purity, Avanti Polar Lipids, Alabaster, AL) (60 mol%) and either ergosterol or cholesterol (both >99% purity, Sigma, St Louis, MO) (40 mol%), and then removing the solvent by evaporation under vacuum overnight. Subsequently, buffer (10 mM Tris, pH 7.4) was added together with 0.1 M carboxyfluorescein (CF) (Sigma, St Louis, MO). After hydration, the lipid mixture was subjected to eight freeze-thaw cycles consisting of freezing in liquid nitrogen and heating to 60°C. Unilamellar liposomes, of about Ø140 nm were generated by multiple extrusions through polycarbonate filters (pore size 100 nm) mounted in a LipoFast miniextruder (Avestin, Ottawa, Canada) at 22°C. Untrapped CF was then removed by two gel filtrations (Sephadex G-50) at 22°C, with Tris buffer as eluent. CF release was determined by monitoring the emitted fluorescence at 520 nm from liposome dispersions (10 mM lipid in 10 mM Tris). An absolute leakage scale was obtained by disrupting the liposomes at the end of the experiment through addition of 0.8 mM Triton X100 (Sigma, St Louis, MO), causing 100% release and dequenching of CF. Although calcein is frequently used for pH-dependent leakage studies, the high charge of this dye has been noted to influence its leakage behaviour in the presence of highly cationic peptides [59]. Instead, therefore, CF was used as a leakage marker at both pH 6.0 and 7.4, however, avoiding pH-dependent fluorescence effects through neutralization prior to probing the limiting leakage in case of pH 6.0 leakage. Throughout, a SPEX-fluorolog 1650 0.22-m double spectrometer (SPEX Industries, Edison, NJ) was used for the liposome leakage assay. Measurements were performed at 37°C.

### CD spectroscopy

The CD spectra of the peptides in solution were measured on a Jasco J-810 Spectropolarimeter (Jasco, U.K.). Measurements were performed at 37°C in a 10 mm quartz cuvet under stirring and the effect on protein/peptide secondary structure monitored in the range 200–260 nm. The background value, detected at 250 nm, was subtracted, and signals from the bulk solution were corrected for. The secondary structure was monitored at a concentration of 0.25 µM of HRG in buffer, in the presence of liposomes (lipid concentration 100 µM), and in the presence of mannan from *Saccharomyces cerevisiae* (0.02 wt%; Sigma-Aldrich, St. Luis, USA).

### Fungal growth in plasma

*C. parapsilosis* ATCC 90018 were grown in TH medium at 27°C to mid-logarithmic phase. The fungi were washed in 10 mM MES, pH 5.5 and resuspended in the same buffer. *C. parapsilosis* (2×10^7^) cfu in a total volume of 10 µl was added to 50 µl of human normal plasma or HRG-depleted plasma (eluent from Ni-NTA agarose gel), and incubated for 0, 4, 8 or 18 hours at 27°C and then plated and number of cfu determined.

### Animal experiments

The original knockout mice 129/B6-*HRG* ^tm1wja1^ were crossed with C57BL/6 mice (Taconic) for 14 generations to obtain uniform genetic background. These HRG-deficient mouse strain was called B6-*HRG* ^tm1wja1^ following ILAR (Institute of Laboratory Animal Resources) rules. Wildtype C57BL/6 control mice and C57BL/6 Hrg^−/−^ mice (8–12 weeks, 27+/−4g) were bred in the animal facility at Lund University. C57BL/6 Hrg^−/−^, lacks the translation start point of exon 1 of the Hrg gene [22]. Animals were housed under standard conditions of light and temperature and had free access to standard laboratory chow and water. In order to study *Candida* dissemination, *C. albicans* ATCC 90018 were grown to midlogarithmic phase, washed and diluted in PBS, pH 7.4. Two hundred and fifty µl containing 1×10^9^ cfu was injected intraperitoneally into C57BL/6 or C57BL/6 Hrg^−/−^ mice, divided into weight and sex matched groups. The animals were sacrificed 48 hours post infection, and blood was collected by cardiac puncture. The number of cfu was determined by viable count. In order to study fungal dissemination to target organs, the mice were infected as previously described and three days later the spleen and kidney were harvested on ice.

### Histology

Representative animals were sacrificed three days post infection and the kidneys were removed into 4% formalin. The tissues were embedded in paraffin, sectioned and stained with Hematoxylin and eosin (H&E) and with Periodic acid-Schiff (PAS).

## Supporting Information

Table S1 Synthetic 20-mer peptides spanning the whole sequence of HRG, used in the screening of antifungal activity in Figure 3B, and relevant descriptive parameters (net charge, activity against *C. albicans* and *C. parapsilosis*, content (%) of the basic amino acids K, R, H, and the acidic D, E.
(0.07 MB DOC)

Figure S1 Correlation between net charge and antifungal activity. 20-mer peptides spanning the whole sequence of HRG (for sequences see Table S1) were used in radial diffusion assay against *C. albicans* ATCC 90028 in 10 mM Tris, pH 7.4 (•) or in 10 mM MES, pH 5.5 (○). A 4 mm diameter well was loaded with 6 µl of 100 µM peptide. The clearance zones (mm) were measured after an overnight incubation at 27°C. The equation for the line of regression is y = 0.605x + 1.222 for peptides in pH 7.4 and y = 0.543x + 0.662 for pH 5.5.
(31.73 MB TIF)

Figure S2 Inhibition of antifungal activity by heparin. 0.3 µM HRG were incubated with 1×10^5^ cfu *C. parapsilosis* ATCC 90018 in 10 mM MES, pH 5.5 with or without 50 µg heparin, plated and the number of cfu determined (n = 6).
(1.16 MB PSD)
