# Supplementary material for: Histidine-Rich Glycoprotein Protects from Systemic Candida Infection
Source: PLoS Pathog. 2008 Aug 1;4(8):e1000116. doi: 10.1371/journal.ppat.1000116 (PMC2537934; doi:10.1371/journal.ppat.1000116)
Supplement: Table S1 — Synthetic 20-mer peptides spanning the whole sequence of HRG, used in the screening of antifungal activity in Figure 3B, and relevant descriptive parameters (net charge, activity against C. albicans and C. parapsilosis, content (%) of the basic amino acids K, R, H, and the acidic D, E. (0.07 MB DOC) [file ppat.1000116.s001.doc]

**Supplementary Table S1.** Synthetic 20-mer peptides spanning the whole sequence of HRG, used in the screening of antifungal activity in Figure 3B, and relevant descriptive parameters (net charge, activity against *C. albicans* and *C. parapsilosis*, content (%) of the basic amino acids K, R, H, and the acidic D, E.

|  |  | net charge  pH 7.4 | net charge pH 5.5 | activity *C. a.* (mm) | | activity *C. p.* (mm) | | K, R (%) | H (%) | D, E (%) |
| --- | --- | --- | --- | --- | --- | --- | --- | --- | --- | --- |
|  |  |  |  | 7.4 | 5.5 | 7.4 | 5.5 |  |  |  |
| 1. | VSPTDCSAVEPEAEKALDLI | - 4.1 | - 3.8 | 0.00±0.00 | 0.00±0.00 | 0.00±0.00 | 0.00±0.00 | 5 | 0 | 25 |
| 2. | DLINKRRRDGYLFQLLRIAD | + 3.0 | + 3.0 | 1.35±.90 | 1.03±0.06 | 1.75±0.48 | 2.74±0.30 | 25 | 0 | 15 |
| 3. | RIADAHLDRVENTTVYYLVL | - 1.0 | - 0.2 | 0.00±0.00 | 0.00±0.00 | 0.00±0.00 | 0.00±0.00 | 10 | 5 | 15 |
| 4. | YLVLDVQESDCSVLSRKYWN | - 1.1 | - 0.9 | 0.00±0.00 | 0.00±0.00 | 0.00±0.00 | 0.00±0.00 | 10 | 0 | 15 |
| 5. | KYWNDCEPPDSRRPSEIVIG | - 1.1 | - 0.9 | 0.00±0.00 | 0.00±0.00 | 0.00±0.00 | 0.00±0.00 | 15 | 0 | 20 |
| 6. | IVIGQCKVIATRHSHESQDL | - 0.1 | + 1.5 | 0.00±0.00 | 0.00±0.00 | 0.00±0.00 | 0.96±0.16 | 10 | 10 | 10 |
| 7. | SQDLRVIDFNCTTSSVSSAL | - 1.2 | - 1.0 | 1.71±.94 | 0.25±0.43 | 2.38±0.62 | 1.24±0.68 | 5 | 0 | 10 |
| 8. | SSALANTKDSPVLIDFFEDT | - 3.0 | - 2.9 | 0.00±0.00 | 0.00±0.00 | 0.00±0.00 | 0.00±0.00 | 5 | 0 | 20 |
| 9. | FEDTERYRKQANKALEKYKE | + 2.0 | + 2.1 | 0.00±0.00 | 2.68±0.28 | 3.83±0.20 | 1.28±0.66 | 30 | 0 | 25 |
| 10. | KYKEENDDFASFRVDRIERV | - 1.0 | - 0.8 | 0.00±0.00 | 0.00±0.00 | 0.00±0.00 | 0.00±0.00 | 25 | 0 | 30 |
| 11. | IERVARVRGGEGTGYFVDFS | 0.0 | + 0.1 | 0.00±0.00 | 0.00±0.00 | 0.00±0.00 | 0.00±0.00 | 15 | 0 | 15 |
| 12. | VDFSVRNCPRHHFPRHPNVF | + 2.0 | + 4.3 | 3.98±0.21 | 5.96±0.13 | 3.85±0.42 | 6.30±0.40 | 15 | 15 | 5 |
| 13. | PNVFGFCRADLFYDVEALDL | - 3.1 | - 2.9 | 0.00±0.00 | 0.00±0.00 | 0.00±0.00 | 0.00±0.00 | 5 | 0 | 20 |
| 14. | ALDLESPKNLVINCEVFDPQ | - 3.1 | - 2.9 | 0.00±0.00 | 0.00±0.00 | 0.00±0.00 | 0.00±0.00 | 5 | 0 | 20 |
| 15. | FDPQEHENINGVPPHLGHPF | - 2.9 | - 0.7 | 0.00±0.00 | 0.00±0.00 | 0.00±0.00 | 0.00±0.00 | 0 | 15 | 15 |
| 16. | GHPFHWGGHERSSTTKPPFK | + 1.1 | + 3.3 | 3.27±0.42 | 5.27±0.57 | 2.80±0.04 | 5.15±0.32 | 15 | 15 | 5 |
| 17. | PPFKPHGSRDHHHPHKPHEH | + 1.1 | + 5.7 | 4.34±0.01 | 5.67±0.22 | 3.07±0.19 | 4.54±0.36 | 15 | 35 | 10 |
| 18. | PHEHGPPPPPDERDHSHGPP | - 2.9 | 0.0 | 0.00±0.00 | 0.00±0.00 | 0.00±0.00 | 0.00±0.00 | 5 | 20 | 20 |
| 19. | HGPPLPQGPPPLLPMSCSSC | - 0.1 | + 0.8 | 0.00±0.00 | 0.00±0.00 | 0.00±0.00 | 0.00±0.00 | 0 | 5 | 0 |
| 20. | CSSCQHATFGTNGAQRHSHN | + 0.9 | + 3.2 | 0.00±0.00 | 0.67±0.59 | 0.00±0.00 | 1.29±0.65 | 5 | 15 | 0 |
| 21. | HSHNNNSSDLHPHKHHSHEQ | - 0.8 | + 4.5 | 0.00±0.00 | 2.74±0.44 | 0.00±0.00 | 2.03±0.48 | 5 | 35 | 10 |
| 22. | SHEQHPHGHHPHAHHPHEHD | - 1.7 | + 5.8 | 0.00±0.00 | 3.90±0.39 | 0.75±0.16 | 4.40±0.55 | 0 | 50 | 15 |
| 23. | HEHDTHRQHPHGHHPHGHHP | - 0.7 | + 6.7 | 0.00±0.00 | 1.58±0.65 | 0.00±0.00 | 5.06±0.33 | 5 | 50 | 10 |
| 24. | GHHPHGHHPHGHHPHGHHPH | + 0.5 | + 8.6 | 3.48±0.32 | 4.59±0.51 | 2.89±0.13 | 4.98±0.15 | 0 | 60 | 0 |
| 25. | HHPHCHDFQDYGPCDPPPHN | - 3.1 | + 1.0 | 0.00±0.00 | 0.00±0.00 | 0.00±0.00 | 0.00±0.00 | 0 | 25 | 15 |
| 26. | PPHNQGHCCHGHGPPPGHLR | - 0.1 | + 3.8 | 1.92±0.27 | 4.70±0.40 | 0.00±0.00 | 4.75±0.31 | 5 | 25 | 0 |
| 27. | GHLRRRGPGKGPRPFHCRQI | + 6.0 | + 7.5 | 7.82±0.71 | 6.78±0.50 | 8.34±0.50 | 6.62±0.26 | 30 | 10 | 0 |
| 28. | CRQIGSVYRLPPLRKGEVLP | + 2.9 | + 3.1 | 2.92±1.11 | 1.59±0.19 | 4.70±0.46 | 3.03±0.38 | 20 | 0 | 5 |
| 29. | EVLPLPEANFPSFPLPHHKH | - 1.0 | + 0.7 | 0.00±0.00 | 0.00±0.00 | 0.00±0.00 | 1.54±0.47 | 5 | 15 | 10 |
| 30. | HHKHPLKPDNQPFPQSVSES | + 0.1 | + 2.5 | 0.00±0.00 | 0.00±0.00 | 0.00±0.00 | 0.00±0.00 | 10 | 15 | 10 |
| 31. | VSESCPGKFKSGFPQVSMFF | + 0.9 | + 1.1 | 0.00±0.00 | 0.00±0.00 | 0.88±0.28 | 1.09±0.23 | 10 | 0 | 5 |
| 32. | GKFKSGFPQVSMFFTHTFPK | + 2.0 | + 2.7 | 2.52±1.00 | 3.68±0.38 | 6.05±0.11 | 4.78±0.34 | 15 | 5 | 0 |
