# Supplementary figures and images for: Histidine-Rich Glycoprotein Protects from Systemic Candida Infection
Source: PLoS Pathog. 2008 Aug 1;4(8):e1000116. doi: 10.1371/journal.ppat.1000116 (PMC2537934; doi:10.1371/journal.ppat.1000116)

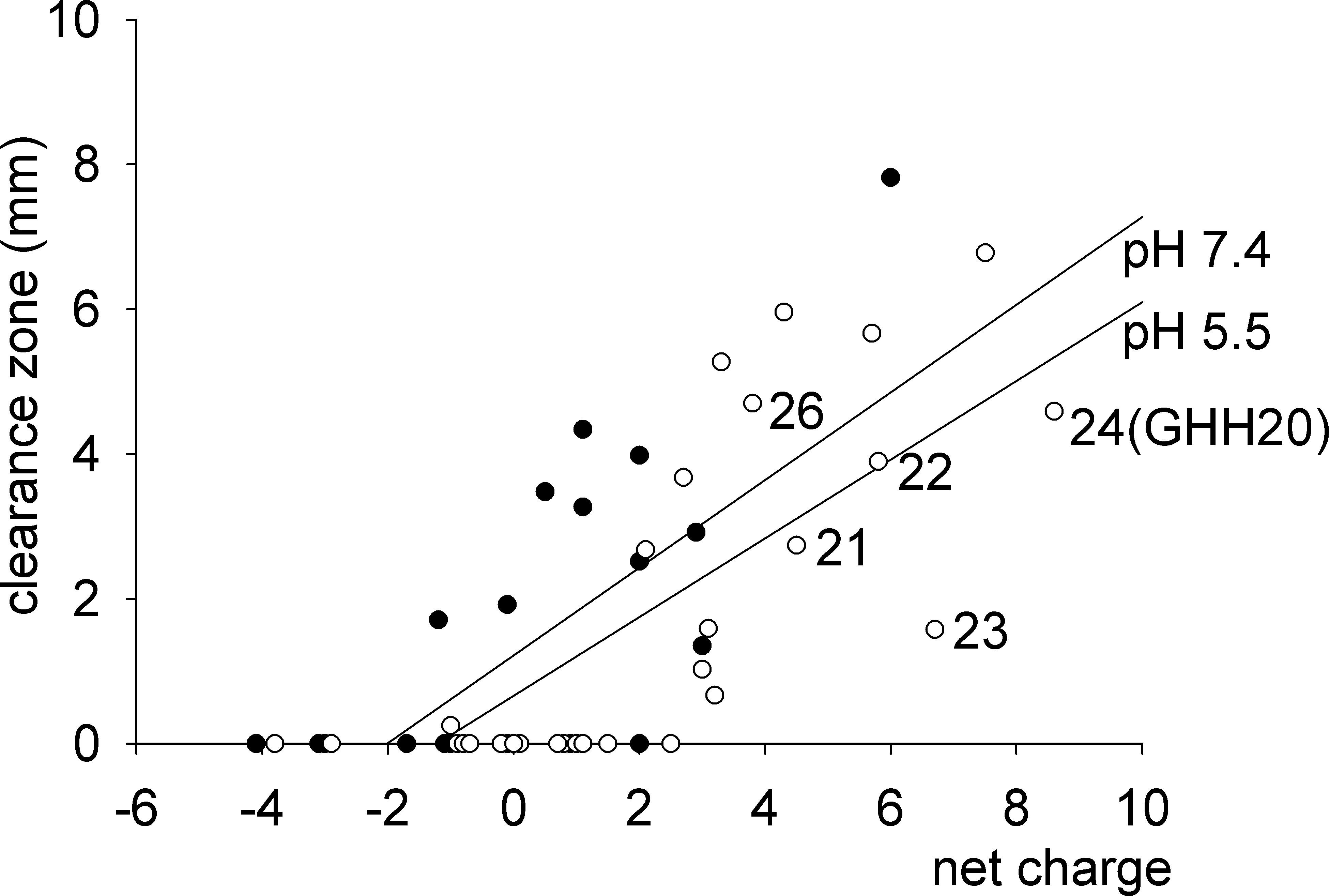

Supplement: Figure S1 — Correlation between net charge and antifungal activity. 20-mer peptides spanning the whole sequence of HRG (for sequences see Table S1) were used in radial diffusion assay against C. albicans ATCC 90028 in 10 mM Tris, pH 7.4 (•) or in 10 mM MES, pH 5.5 (○). A 4 mm diameter well was loaded with 6 µl of 100 µM peptide. The clearance zones (mm) were measured after an overnight incubation at 27°C. The equation for the line of regression is y = 0.605x + 1.222 for peptides in pH 7.4 and y = 0.543x + 0.662 for pH 5.5. (31.73 MB TIF) [file ppat.1000116.s002.tif]
